# Rhizospheric* Bacillus subtilis* Exhibits Biocontrol Effect against* Rhizoctonia solani* in Pepper* (Capsicum annuum)*

**DOI:** 10.1155/2017/9397619

**Published:** 2017-12-28

**Authors:** Yuanyuan Huang, Zhansheng Wu, Yanhui He, Bang-Ce Ye, Chun Li

**Affiliations:** School of Chemistry and Chemical Engineering, The Key Laboratory for Green Processing of Chemical Engineering of Xinjiang Bingtuan, Shihezi University, Shihezi 832003, China

## Abstract

This study aimed at evaluating the ability of SL-44 to control* Rhizoctonia solani* and promote pepper* (Capsicum annuum)* growth. Strain SL-44 was isolated from plant rhizosphere and the pot experiment results indicated that the dry and fresh weights of pepper in SL-44 and* Rhizoctonia solani* (S-R) treatment were 45.5% and 54.2% higher than those in* Rhizoctonia solani* (R) treatment and 18.2% and 31.8% higher than those in CK (control, noninoculation) treatment. The plant height in S-R treatment increased by 14.2% and 9.0% compared with those in the R and CK treatments, respectively. In vitro antagonism assay showed that SL-44 exhibited strong antifungal activity against the mycelial growth of* Rhizoctonia solani*, with an inhibition rate of 42.3%. The amount of phosphorus dissolved by SL-44 reached 60.58 mg·L^−1^ in broth and 7.5 *μ*g·mL^−1^ IAA were secreted by SL-44. Strain SL-44 inhibited the growth of* R. solani* and improved biomass of pepper plants. Mass exchange and information transmission between the pepper plants and SL-44 mutually promoted their development.* Bacillus subtilis* SL-44 has a great potential as biocontrol agent against* Rhizoctonia solani* on pepper plants.

## 1. Introduction

Plant growth-promoting rhizobacteria (PGPR) are beneficial microorganisms that live in soil and colonize the rhizosphere. These microorganisms can promote plant growth and facilitate the absorption and utilization of mineral nutrients [1, 2]. PGPR can increase the crop yield and reduce disease occurrence and thus is considered the most promising agent for cash crop production [3]. Generally, PGPR are involved in many species, such as* Pseudomonas*,* Bacillus*,* Agrobacterium*,* Erwinia*,* Flavobacteria*,* Pasteuria*,* Enterobacter*,* Azospirillum*, and* Hafnia* [4].* Bacillus subtilis* HS93 and* Bacillus licheniformis* LS674 were isolated from roots of pepper plants; the antagonistic activity of two bacteria may be stimulated by chitin resulting in significant improvements in their effectiveness against* Rhizoctonia solani* and* Phytophthora capsici *[5].* Bacillus simplex* RC19, a growth-promoting bacterial species in the plant rhizosphere, produces indole-3-acetic acid (IAA) for treatment of kiwifruit cuttings and significantly promoting rooting and root growth [6].

PGPR act on plants through two aspects: some PGPR can synthesize auxin and siderophore that can immediately promote plant growth, and unabsorbable nutrient elements were transformed into absorbable by PGPR that benefit plant nutrient uptake (including phosphate solubilization and nitrogen fixation). PGPR are involved in phosphorous solubilization. Panhwar et al. [7] isolated phosphorus-solubilizing bacteria (PSB) from the rhizosphere soil of aerobic rice, the PSB exhibited phosphate-solubilizing activity up to 69.58% and produced siderophore and organic acids, and the PSB also produced secondary metabolites, which can promote the growth and development of plants. Moreover, PGPR can secrete phytohormones, such as IAA, which can stimulate plant growth [8].

Root exudates are complex and heterogeneous systems that are released from different parts of the root to the growth matrix during the growth process. Root exudates in the rhizosphere act as a source of nutrients for other organisms [9]. Plant root exudates may provide sugars, amino acids, and vitamins for the growth and reproduction of rhizobacteria and may influence the type and quantity of soil microorganisms in the rhizosphere [10].

Infection of pathogenic fungi (particularly* Rhizoctonia solani*) is a significant factor that causes disease and decreases the yield of pepper* (Capsicum annuum)* plant [11]. Pathogenic fungi produce mycelia or spores, which spread in the soil and damage the growth of host plants. Biological control of plant diseases has gained attention due to increased pollution concerns caused by excessive use of pesticides for crop protection and development of pathogen resistance [12]. To prevent and control plant soil-borne diseases, scholars have explored environment-friendly methods for biological control of plant diseases. For example, microorganisms can be used to effectively promote plant growth due to their role in nutrient cycling [13].

Many researches showed that* Bacillus* strains can be biological control agents against* Rhizoctonia solani* infecting pepper [5], potato [14, 15], tomato [16] plant, and so forth. Moreover,* Bacillus subtilis* has been widely studied for its mechanisms against various plant disease due to its ability to produce antibiotics, lipopeptides, and hydrolytic enzymes [15, 17]. A complex response with a range of antagonistic effects among* Bacillus *strains and distinct responses by different pathogens has been observed by researchers; it could be expected as a novel mechanism [14].

In this study,* Bacillus subtilis* SL-44 was isolated from plant rhizosphere and tested for antagonistic activity on the pathogen* R. solani* in laboratory. The P solubilization and capability for IAA production of the strain were investigated. Pepper seedling growth conditions were estimated, and the effects of SL-44 on root colonization were studied. Chemotactic response caused by pepper root exudates was also evaluated. Results provide a basis for using PGPR to prevent pathogens and promote plant growth. Hence, SL-44, which is a nonpollutant, can be used in disease biocontrol and plant growth promotion in the future.

## 2. Materials and Methods

### 2.1. Biocontrol Strain and Phytopathogen

The plant fungal pathogen* R. solani* was donated by the Key Laboratory of Crop Disease Control and Prevention of Shihezi University.* B. subtilis* SL-44 was isolated from rhizosphere soil of cotton. Pepper seeds, wheat bran, and vermiculites were acquired from a local market in Shihezi, Xinjiang province.

### 2.2. Bacterial Characterization and 16S rDNA Identification

SL-44 was characterized in morphological, physiological, and biochemical characterization and then compared with standard species using Bergey's Manual of Determinative Bacteriology. The bacterial isolate was genetically identified by 16S rDNA gene sequence analysis; the PCR amplification was performed using universal primers according to the method of Yao et al. [18]. The amplification products were purified using a PCR purification kit (Tiangen, China) and sequenced.

### 2.3. In Vitro Antagonism Activity Test

Antifungal activity was evaluated by dual culture method [19].* R*.* solani* were propagated to test antagonistic effects of SL-44 on the center of potato dextrose agar (PDA) medium (containing the extract of 200 g of boiled potatoes, 20 g of dextrose, and 20 g of agar in 1000 mL of distilled water, pH 7.0–7.2). 100 uL SL-44 (10^8^ CFU·mL^−1^) bacterial suspension was inoculated on the PDA plate and incubated at 30°C. Equal volume of sterilized water was used as control treatment. Inhibition zone was observed after 3–5 days, and the diameter was recorded. The experiments were repeated three times. Inhibition rate (IR%) was calculated to determine the inhibitory effect of SL-44 using the following equation:

(1) $$\begin{array}{c} IR\left(\;\%\;\right)=\frac{\left(C-B\right)}{C}\times 100, \end{array}$$

where *C* is the diameter of the control fungal mycelium and *B* is the diameter of the fungal mycelium grown in the presence of the bacteria [15].

### 2.4. Siderophore Generation by SL-44

Siderophore production was detected by the classical method established by Schwyn and Neilands [20]. The generated siderophore was evaluated on chrome azurol S (CAS) agar plates. Siderophore production was assessed by a yellow halo generated around colonies. 10 *μ*L (10^11^ CFU·mL^−1^) of SL-44 was added to surface of the medium; an equal volume of CK1 (sterilized distilled water) and CK2 (inactivated bacteria) was used control. The color variation was observed after three days of incubation at 28°C. The experiments were repeated three times.

### 2.5. Nitrogen Fixation Property of Bacteria

Qualitative detection was adopted to estimate the nitrogen fixation performance of SL-44 by using nitrogen fixation test medium (NFb) [21]. The bacterial broth cultured to logarithmic growth phase (10 *μ*L) was dripped to the NFb plate and cultured at 28°C for 48 h. Inactivated bacterial broth was set as control (CK). The experiments were repeated three times.

### 2.6. P Solubilization in Liquid Cultures

The content of soluble P in the bacterial suspension was determined according to molybdate blue colorimetric method [22]. Briefly, 1% of SL-44 inoculum was added to 30 mL of Pikovskaya medium (PKO) (containing 10 g of glucose, 10 g of Ca_3_(PO_4_)_2_, 0.5 g of (NH_4_)_2_SO_4_, 0.3 g of NaCl, 0.3 g of KCl, 0.3 g of MgSO_4_·7H_2_O, 0.03 g of FeSO_4_·7H_2_O, 0.03 g of MnSO_4_·4H_2_O, and 1000 mL of H_2_O; pH 7.0–7.5) and incubated in a shaker at 30°C and 180 rpm under continuous culturing of 60 h. The pH and soluble P concentration were determined by using a pH meter and colorimetric method, respectively. The experiments were repeated three times.

### 2.7. Ability of SL-44 to Secrete IAA

The IAA production capacity of SL-44 was quantitatively estimated with Salkowski reagent by using the method reported by Glickmann and Dessaux [23]. SL-44 was inoculated in Luria–Bertani liquid medium (10 g of peptone, 5 g of NaCl, 10 g of yeast extract, and 1000 mL of H_2_O; pH 7.0–7.2; added with 100 mg·L^−1^ L-tryptophan) and incubated at 180 rpm and 30°C for 60 h. Briefly, 5 mL of the supernatant and an equal volume of Salkowski reagent (10 mL of 0.5 M FeCl_3_, 500 mL of 35% HClO_4_) were mixed for colorimetric assay.

### 2.8. Pot Experiment Design

Pepper seeds were sterilized by immersion in 0.1% HgCl_2_ for 5 min and in 75% ethanol for 2 min and washed four times with sterile water. Prepared pepper seeds were sowed randomly in plastic pots (9 cm diameter; 12 cm depth) containing 200 g of the mixture matrix (volume ratio of vermiculite and pearlite, 6 : 4; vermiculite diameter, 0.1–0.3 mm). Four treatments were prepared: S, pepper inoculated with* Bacillus subtilis* SL-44; S-R, pepper inoculated with* Bacillus subtilis* SL-44 and* R. solani*; R, pepper inoculated with* R. solani*; CK, noninoculation. Each treatment was performed in triplicate. The pathogenic* R. solani* was propagated in wheat bran medium. Plants in all the treatments were grown in a growth chamber at 30°C with a 12 h light/12 h dark photoperiod. Water and Hoagland nutrient were used to sustain normal plant growth.

Twenty-day-old plants were inoculated with SL-44 and* R*.* solani *to investigate bacterial colonization on the pepper root surface. Pepper root samples were collected (three plants per replication) after 5, 10, and 15 days of injection. Bacteria were extracted from 0.2 g of roots randomly selected from each treatment by removing vermiculites that adhered on the roots. The roots were immersed in 50 mL of PBS buffer (pH 7.0) and shaken for 0.5 h. Colony counting method was used to measure the colony forming units of bacteria.

### 2.9. Root Exudate Collection and Chemotaxis Assay

Root exudates were collected according to the root-submerged method proposed by Badri et al. [24] with some modifications. “Drop” assay method described by Yuan et al. [9] was applied for qualitative chemotaxis assay. SL-44 was grown in NA medium at 37°C and 170 rpm until an OD_600_ of 0.8 was reached. Cells grown in 50 mL of the medium were collected by centrifugation and resuspended in 12 mL of chemotaxis buffer (100 mM potassium phosphate [pH 7.0] with 20 *μ*M EDTA). Briefly, 4 mL aliquot of 1% hydroxypropylmethylcellulose solution was added to the cell suspension. The concentrated root exudates were added to the center of each Petri dish. A ring of turbid zone near the center of each Petri dish appeared after 10–15 min of incubation at room temperature (RT) if the chemotactic response of bacterial cells was triggered.

### 2.10. Gas Chromatography-Mass Spectrometry (GC-MS)

Briefly, 1 *μ*L of each sample was injected into an Agilent 6890N GC coupled with a 5973 MS to analyze chemical compounds in the root exudates. A DB5-MS (J & W Scientific) 60 m capillary column (30 m × 0.25 mm, 0.25 *μ*m film thickness) was used to achieve the required chromatographic condition, and helium was applied as carrier gas at a flow rate of 1 mL·min^−1^. The injector temperature was maintained at 250°C, and the temperature program was set as follows: column temperature was held at 50°C for 2 min, ramped at 6°C·min^−1^ to a final temperature of 250°C, and held for 15 min. The conditions of the mass spectrum included the following: electron impact ion source, EI; ion energy, 70 eV; ionization temperature, 200°C; scanning range, 30–600 amu; and sample volume, 1 *μ*L, with splitless injecting samples.

### 2.11. Statistical Analysis

Data were tested for statistical significance using the analysis of variance package included in Microsoft Excel 97, and comparison was done using a least significant difference (LSD) test (*P* = 0.05).

## 3. Results 

### 3.1. Identification of SL-44

The characteristics of SL-44 colonies are described in Table 1. The morphology of the SL-44 colony was opaque, rough, and wrinkled;* B. subtilis* was used as control strain. SL-44 was tested positive for gram stain, oxidase, glucose, V-P, starch hydrolysis, and citrate utilization but tested negative for arginine hydrolysis and fluorescence. The determined properties of SL-44 are in agreement with the description of* B. subtilis*. Thus, SL-44 was preliminarily identified as* Bacillus subtilis*.

The strain SL-44 was found to be approximately 1541 bp in size and deposited in the GenBank nucleotide database (accession number FJ788428) at the NCBI website. Analysis using the BLASTn program indicated that SL-44 shared a close relationship as 100% similarity to the DNA sequence of* Bacillus subtilis*.

### 3.2. In Vitro Pathogenic Fungi Inhibition Efficiency

**Figure 1:**
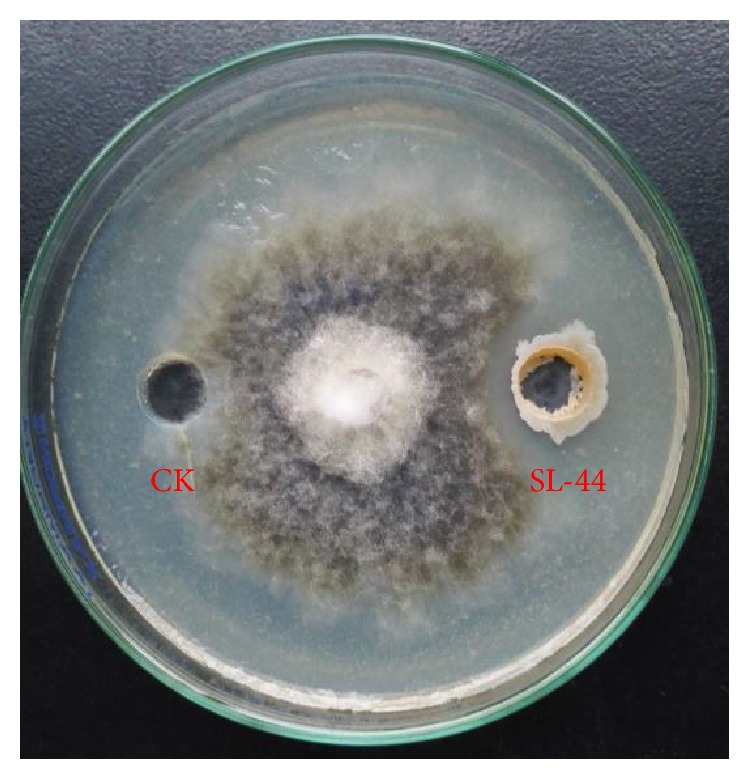
Inhibition effect of antagonistic strain SL-44 on* R. solani*. CK, sterilized water; SL-44, bacterial suspension (10^8^ CFU·mL^−1^).

The antifungal activity of the strain SL-44 against* R. solani* was examined by dual culture assay in PDA plates. The inhibition zone was clearly observed. SL-44 showed strong antifungal activities against the mycelial growth of* R. solani*, with an inhibition rate of 42.3% relative to that of CK (Figure 1).

### 3.3. Bacterial Dissolution of Phosphate in Continuous Culture Medium

**Figure 2:**
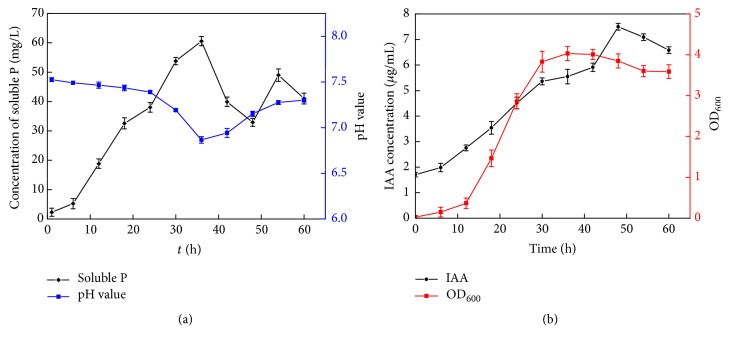
Concentration variation of dissolved P (a) and IAA (b) of SL-44 broth. Error bars indicate standard deviation.

The dynamic concentration of soluble P for* B. subtilis* SL-44 was investigated in continuous culture medium for 60 h (Figure 2(a)). The concentration of soluble phosphate increased within the first 12 h of incubation, whereas the pH of the culture medium slightly decreased. The maximum concentration of soluble P in the SL-44 broth was 60.58 mg·L^−1^ as the pH of the culture medium decreased to 6.86 after 36 h of incubation. This finding indicates the inverse correlation of the pH of the SL-44 liquid broth with the concentration of dissolved P. The amount of dissolved phosphate rapidly decreased to 32.93 mg·L^−1^ after 48 h of incubation. However, the soluble P content slightly increased in the decline phase of cell growth.

### 3.4. Production of IAA by SL-44

The IAA yield continuously increased under prolonged culture in the LB medium (Figure 2(b)). The IAA level peaked at 7.50 *μ*g·mL^−1^ when the strain was incubated for 48 h. SL-44 had abundant biomass and synthesized IAA in the late stationary growth phase. Thereafter, the IAA content decreased.

### 3.5. Effect of SL-44 on the Activities of Siderophore

**Figure 3:**
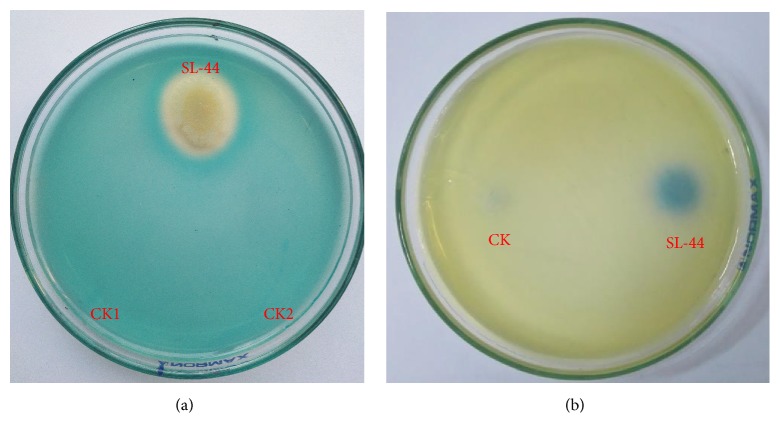
Effect of siderophore secretion (a) and nitrogen fixation (b) by SL-44. (a) CK1, sterilized distilled water; CK2, inactivated bacteria; (b) CK, inactivated bacterial suspension.

The color of SL-44 treatment markedly changed from blue to yellow compared to CK1 and CK2 (Figure 3(a)). The diameter of halo was 2.5 cm. However, there is no halo generated around CK1 and CK2 treatments. The result showed SL-44 with ability of siderophore production.

### 3.6. Nitrogen-Fixing Character of Strain SL-44

There was an obvious change in color varied from yellow to blue around SL-44 treatment (Figure 3(b)), and diameter of the halo was found to be 1.8 cm, whereas there were no color changes around CK. Accordingly, nitrogen fixation was proved by SL-44.

### 3.7. Plant Growth Index

**Figure 4:**
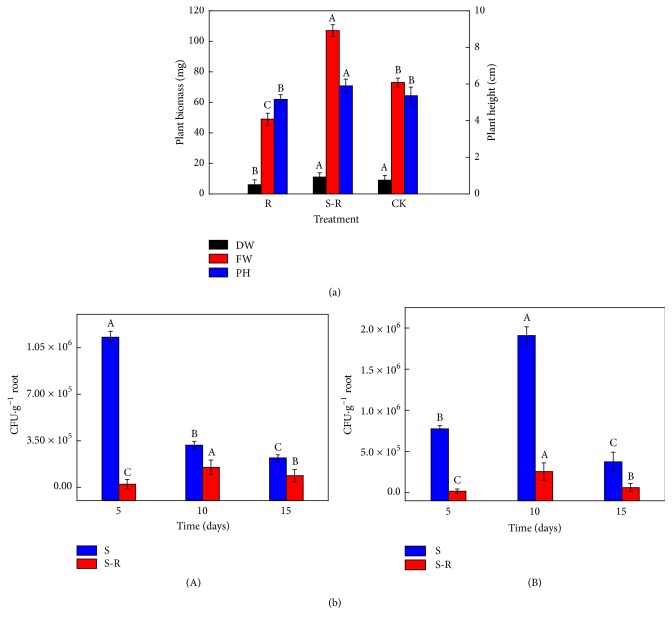
Growth indices of pepper plants under different treatments (a) and colonization density of SL-44 (b). (a) DW, dry weight; FW, fresh weight; PH, plant height; (b) 0–2 cm (A) and 2–4 cm (B) pepper root length. A, B, and C indicate a significant difference at *P* < 0.05 according to LSD.

SL-44 inoculation was used to evaluate the effect of the bacterium on promoting pepper plant growth. Plant growth parameters including fresh weight, dry weight, and individual plant height were measured after 25 days of sowing in the three treatments (Figure 4(a)). The indices significantly increased in S-R treatments compared with R and CK. The dry weight of S-R treatment was 45.5% and 18.2% higher than that in R and CK treatments, respectively. The fresh weight of S-R treatment was 54.2% and 31.8% higher than that in R and CK treatments, respectively. The fresh weight of the plant showed the highest significant difference among S-R and the other treatments. In addition, the plant height of S-R treatment was increased by 14.2% and 9.0% compared with those in R and CK treatments, respectively.

### 3.8. Colonization of SL-44 Strain

The results of SL-44 colonization assay were showed in Figure 4(b). Bacterial colonization in S treatment decreased with increasing inoculation time in the 0–2 cm depth, when reached 15 days after inoculation; the amount of SL-44 was 2.2 × 10^5^ CFU·g^−1^ root [Figure 4(b)(A)]. However, the amount of colonization increased first and then decreased in S-R treatment, and the maximum of colonization was 1.5 × 10^5^ CFU·g^−1^ root after 10 days of inoculation.

The colonization of SL-44 in the S and S-R treatments was investigated in the 2–4 cm depth [Figure 4(b)(B)]. The maximum value of colonization was 1.9 × 10^6^ CFU·g^−1^ root and 2.5 × 10^5^ CFU·g^−1^ root in S treatment and S-R treatment after 10 days of inoculation, respectively. The colonization quantity of SL-44 tended to increase first and then decrease with increasing inoculation time both in S and in S-R treatments [Figure 4(b)(B)].

### 3.9. Chemotactic Response of SL-44 to Pepper Root Exudates

**Figure 5:**
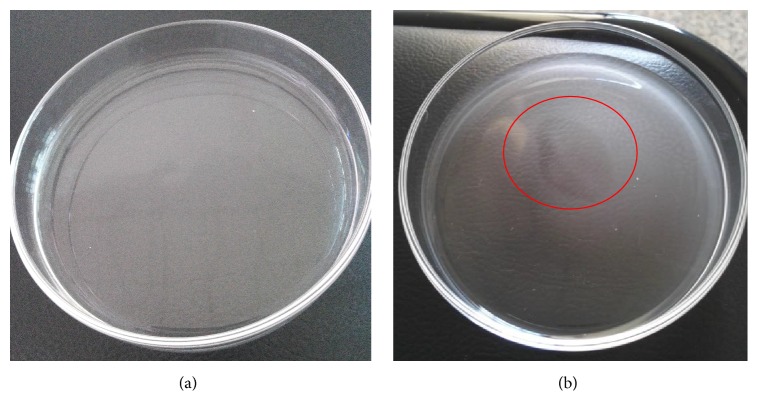
Chemotactic response of* B. subtilis *SL-44 toward pepper root exudates. (a) Chemotaxis buffer; (b) concentrated root exudates of pepper.

In the qualitative assay, the concentrated root exudates were attracted to higher number of cells in SL-44 compared with that in the control (no root exudates) (Figure 5). A clear ring of turbidity was found near the center of the Petri dish. The chemotactic response of SL-44 might be induced in the presence of pepper root exudates.

### 3.10. Analysis of Root Exudates by GC-MS

The root exudates of pepper contain eighteen different organic compounds, namely, seven hydrocarbons, six esters, four aromatic hydrocarbons, and one organic acid (Table 2).

## 4. Discussion

In many studies,* Bacillus subtilis* strains had proved its high efficiency on control* Rhizoctonia solani*, produced IAA, siderophore, and nitrogen fixation to promote plant growth [16, 25].

The physiological and biochemical properties, 16S rRNA sequence analysis, and the high similarity values confirmed that SL-44 and* B. subtilis* belong to the same species [26].* Rhizoctonia solani* was inhibited when SL-44 compete with it for nutrients and space on the PDA plate. The inhibition effect of SL-44 could be due to lytic enzymes such as glucanase, chitinase, and protease which were produced, which directly degrade the cell walls of pathogenic fungi, and lipopeptides like iturin and fengycin produced by antagonistic bacteria can be directly against or kill phytopathogens [17, 19, 27].

Content of soluble P in SL-44 culture medium was varied during 60 h of incubation. The increase of P-solubilized concentration could be due to the consumption of glucose from the growth media and the release of organic acids, such as gluconic acid, from SL-44. Similar results were extrapolated from a previous study [28].* Pseudomonas fluorescens *strains L228 and L132 exhibited high potential of P solubilization, with content of soluble P exceeding 1000 mg·L^−1^; gluconic acid was also produced and detected in the medium as the pH of the medium decreased [29]. The reduction of the quantity of soluble phosphate after reaching the highest level of production during incubation can be explained by the self-consumption of soluble phosphate by the growing bacterial population [30]. The ability of the strain to release P was evaluated based on the levels of elements liberated, which were either dissolved in the supernatant in the form of orthophosphate or were assimilated by microorganisms to form cell biomass [31]. The soluble P content slightly increased in the decline phase of cell growth because self-decomposition could lead to the release of P from the cell. Meanwhile, the soluble phosphate precipitated into its insoluble state. Similarly, Peng et al. [32] reported that* P. putida* Rs-198 exhibiting phosphate-solubilizing ability led to varied P concentration ranging from 65 *μ*g·mL^−1^ to 118 *μ*g·mL^−1^ in the medium at stationary growth phase. Mardad et al. [31] isolated* Enterobacter hormaechei* subsp. strain PSB 6 that can strongly solubilize P, ranging from 400 mg·L^−1^ to 505 mg·L^−1^ after 36 h to 72 h of incubation. The phosphate solubilization ability of SL-44 may promote nutrient uptake and growth of plants.

Siderophore production helps a particular microorganism to compete effectively for available iron against other organisms. The production of siderophore is one of the most common bacterial strategies for acquiring iron under iron-limited conditions. Yu et al. [33] isolated a kind of siderophore-producing strain; namely,* Bacillus subtilis* CAS15 reduced the incidence of* Fusarium wilt* in pepper significantly, by 12.5–56.9%. Sadeghi et al. [34] reported that siderophore-producing* Streptomyces* strain C enhanced iron acquisition and wheat growth promotion under saline condition. SL-44 was able to grow in the nitrogen-free culture medium possibly because of the acquisition of atmospheric nitrogen by biological fixation. The changes in the color of the medium could be due to variations in pH when N_2_ in the air was transformed by the strain into ammonium compounds. The pH of the medium also turned alkaline. In addition, the color of the NFb medium changed to blue [21]. Hence, SL-44 may exhibit significant ability in nitrogen fixation for promoting plant growth. Similarly, Çakmakçı [25] has screened some rhizobacteria isolates, which have abilities of in vitro plant growth-promoting traits such as production of IAA, nitrogenase activity, and phosphorus (P) solubilization. Chaiharn and Lumyong [35] reported that the IAA level produced by* Klebsiella* SN 1.1 reached 790 *μ*g/mL (maximum) at the stationary phase but decreased in the growth medium during the late stationary phase of growth. Shao et al. [2] reported similar conclusions on IAA production by* Bacillus amyloliquefaciens* SQR9; the IAA concentration reached 9.46 mg·L^−1^ in the supernatant at stationary phase and decreased in the broth during the late stationary phase of growth (80 h).

Pot experiment showed that SL-44 indeed play a significant role in promoting pepper growth. These findings could be due to the biocontrol properties of SL-44. Hence, SL-44 may improve plant growth and increase plant biomass by several mechanisms, such as production of IAA and siderophore. Luo et al. [36] determined the effects of a plant growth-promoting endophyte,* Bacillus sp*. SLS18; the strain produced IAA and siderophores and influenced the biomass production of sweet sorghum (*Sorghum bicolor *L.). In addition, the nitrogen fixation of SL-44 promoted the nitrogen nutrient absorption of the plant. Taurian et al. [37] have reported that the P-solubilizing strain* Pantoea* J49 has the ability of increasing the peanut biomass which could be related to mechanisms such as nitrogen fixation and IAA production. Moreover, SL-44 with antagonistic ability in vitro was confirmed to inhibit the mycelium growth of* R. solani *and directly promote plant growth. Some researchers have found that* B. subtilis* showed significant inhibitory effects on the growth of* R. solani* by reduced disease incident and promoted plant growth [14, 16].

Based on the ability of colonization of SL-44, the results of 0–2 cm colonization can be explained based on root system upgrowth, where the roots become longer and more robust; the bacteria searched for nutrients from deeper roots, leading to decreased colonization with increasing time in S treatment, while the variation of bacterial colonization in S-R treatment may be due to the changed growth condition of SL-44, which takes time to adjust and adapt to the living environment. When the strain adapted to the environment, root exudates and exfoliated root sheath cells can serve as sources of nutrients for microorganisms. SL-44 can exchange information and substances with the roots, thereby colonizing on plant roots stably. However, root exudates and exfoliated root sheath cells cannot be unique nutrients for bacterial growth and reproduction because they cannot satisfy all substances required for the growth of microorganisms.

The amounts of SL-44 colonization were increased in first 10 days of inoculation with the development of pepper roots at 2–4 cm root length. However, root exudates and exfoliated root sheath cells cannot be unique nutrients for bacterial growth and reproduction because they cannot fulfill entire substances required for the growth of microorganisms. As such, the number of viable SL-44 decreased after 15 days of inoculation. The less colonization in the S-R treatment than that in S treatment might be due to the competition between* R. solani* and SL-44 for spaces and nutrients (S-R treatment). Thus, the number of SL-44 in the roots slightly decreased. Analysis of root colonization within the depths of 0–2 and 2–4 cm showed that although, with the existence of* R. solani*, SL-44 can compete with them for spaces and nutrients, it also plays an important role in promoting the growth of pepper plant. Hence, SL-44 may protect pepper from* R. solani* and exhibit high colonization ability. This finding provides a basis for studies on the interaction between strains and plants.

Bacteria eliciting positive chemotactic effect on a chemotactic attractant will move toward chemotactic substances; as such, the concentration of bacteria in the droplets of chemotactic substances will be higher than the external concentration. Furthermore, a transparent ring was formed at the edge of the droplet [9]. Other studies highlighted the role of root exudates in PGPR colonization on roots. Ling et al. [38] reported that the root exudates of watermelon recruited the beneficial rhizobacterium* P. polymyxa* SQR-21 and promoted bacterial colonization. Root exudates induce certain beneficial microorganisms to gather and colonize the rhizosphere. Chemotaxis toward root exudates is the first step of bacterial colonization. Previous studies showed that the root exudates of* Capsicum* mainly contain acids, ketones, amines, esters, alcohols, phenols, hydrocarbons, benzene, and other heterocyclic compounds. Heterocyclic compounds exert many biological activities, such as insecticidal effect, plant growth regulation, and other functions [10].

SL-44 was identified as* B. subtilis* based on the characterization of its physiology, biochemistry, and 16S rDNA sequence and regarded as potential agent for biocontrol and growth promotion. SL-44 elicited strong inhibitory effect on* R. solani*. Pot experiment results indicated that SL-44 effectively enhance the biomass and height of pepper seedlings. Moreover, SL-44 exhibited the ability of nitrogen fixation, leading to abundant soluble P and nitrogen that can be absorbed by plants for growth. Furthermore, SL-44 secreted IAA, which can stimulate the growth and development of pepper. The results of colonization and chemotaxis tests showed that SL-44 survived stably in the rhizosphere and interacted with plants. As such, mass exchange and information transmission between the plants and microorganisms mutually promoted their development. Therefore, SL-44 is a suitable agent for biocontrol and plant growth promotion.

## Figures and Tables

**Table 1:** Physiological and biochemical properties of SL-44.

Characteristic	SL-44	*B. subtilis*
Colonial morphology	wrinkle; rough; opaque	wrinkle; rough; opaque
Colonial color	ivory-white	ivory-white
Cell shape	Rod	Rod
Germ stain	+	+
Gram stain	+	+
Fluorescence	−	−
Oxidase	+	+
Catalase	+	+
Glucose	+	+
Voges-Proskauer's	+	+
Starch hydrolysis	+	+
Nitrate reduction	+	+
Citrate utilization	+	+
Gelatin liquefaction	+	+
Arginine hydrolysis	−	−

+: positive; −: negative.

**Table 2:** GC-MS identification of main substances in the root exudates of pepper.

Sequences	Retention time	Similarity	Molecular formula	Chemicals
(1)	3.145	91	C_2_H_4_O_2_	Acetic acid
(2)	3.449	80	C_4_H_9_NO	Acetic anhydride
(3)	3.857	90	C_7_H_8_	Toluene
(4)	3.895	91	C_7_H_8_	1,3,5-Cycloheptatriene
(5)	4.303	80	C_6_H_12_O_2_	Butanoic acid, ethyl ester
(6)	4.503	78	C_6_H_12_O_2_	Acetic acid, butyl ester
(7)	5.351	94	C_8_H_10_	Ethylbenzene
(8)	5.519	97	C_8_H_10_	Benzene, 1,3-dimethyl-
(9)	5.972	97	C_8_H_10_	o-Xylene
(10)	13.133	94	C_12_H_26_	Dodecane
(11)	14.996	76	C_11_H_24_O	Tridecane, 4-methyl-
(12)	17.635	97	C_14_H_30_	Tetradecane
(13)	18.851	93	C_10_H_10_O_4_	Dimethyl phthalate
(14)	21.678	95	C_16_H_34_	Hexadecane
(15)	22.984	91	C_12_H_20_O_7_	Triethyl citrate
(16)	25.32	94	C_9_H_19_I	Octadecane
(17)	28.082	94	C_16_H_22_O_4_	Dibutyl phthalate
(18)	28.631	87	C_20_H_42_	Eicosane
